# Seasonal patterns in Bell's palsy: a systematic review and meta-analysis

**DOI:** 10.3389/fneur.2025.1626018

**Published:** 2025-11-11

**Authors:** Bander Fahad Aljarallah, Faisal Sulaiman Alolayqi, Bassam Ahmed Alghizzi, Khalid Ahmed Alomari, Salem Khalaf Alanazi, Thamer Ali Alharbi, Asma Saleh Alshahrani, Aljoharah A. Alshaikh, Makki Ahmed Almuntashri

**Affiliations:** 1 King Saud bin Abdulaziz University for Health Sciences, Riyadh, Saudi Arabia; 2 King Abdullah International Medical Research Center, Riyadh, Saudi Arabia; 3 King Abdulaziz Medical City, Ministry of National Guard Health Affairs, Riyadh, Saudi Arabia; 4 College of Medicine, King Saud University, Riyadh, Saudi Arabia; 5 Department of Population Health, King Abdullah International Medical Research Center, Riyadh, Saudi Arabia

**Keywords:** Bell's palsy, seasonality, idiopathic facial paralysis, systematic review, meta-analysis

## Abstract

**Background:**

Bell's palsy (BP) is an idiopathic condition affecting the seventh cranial nerve, causing unilateral facial muscle paralysis. Conflicting reports exist on the impact of seasonal variations on BP, and limited systematic reviews have been conducted. In this study, a systematic review and meta-analysis of the BP literature was performed to assess the association between BP and the four seasons.

**Methods:**

In accordance with PRISMA guidelines, a systematic search was performed using the PubMed, Google Scholar, and Web of Science databases to identify cohort studies reporting seasonal cases of idiopathic BP. Study quality and risk of bias were assessed via the Newcastle–Ottawa Scale (NOS). The associations between seasonal variations and the occurrence of BP were analyzed via a random effects model.

**Results:**

Eight cohort studies involving 3,363 BP patients were included, with a slight male predominance (51.5%). The pooled mean age was 44.72 ± 19.6 years. Pooled proportions for each season were determined via subgroup analysis. Winter proportions were the highest at 0.27 (95% CI: 0.24–0.31; *I*^2^ = 65.6%, *p* = 0.0048), followed by autumn at 0.26 (95% CI: 0.23–0.29; *I*^2^ = 59.3%, *p* = 0.0161). The spring proportion was 0.24 (95% CI: 0.20–0.27; *I*^2^ = 68.1%, *p* = 0.0026), whereas the summer proportion was 0.22 (95% CI: 0.19–0.25; *I*^2^ = 61.5%, *p* = 0.0111). The test for subgroup differences showed (χ^2^ = 6.62, *p* = 0.0850).

**Conclusion:**

BP cases were more common during colder months than during warmer months. Although no statistically significant association was found, this highlights the need for further studies across diverse climatic regions to clarify potential seasonal influences.

**Systematic review registration:**

https://www.crd.york.ac.uk/PROSPERO/view/CRD42024623519, identifier: CRD42024623519.

## 1 Introduction

Bell's palsy (BP), also known as idiopathic facial paralysis (IFP), is an acute lower motor paresis of the seventh cranial nerve (CN VII). BP has been established as the leading cause of facial paralysis (1, 2). Damage to the motor nerve fibers of CN VII might result in complete or partial dysfunction of the facial musculature (2). House-Brackmann Grading (HBG) scale is a subjective grading system that involves observing muscle movement to assess the degree of facial muscle functionality and recovery (3). Currently, there are no established etiologies accounting for BP development; however, inflammatory processes or hypercoagulability could lead to facial nerve injury (4, 5). Acute oral herpes simplex virus type 1 (HSV-1) has been shown to cause facial paralysis in lab rats when the virus invades subcutaneous tissue, with facial nerve demyelination as the underlying mechanism (6). It has also been identified that reactivation of latent varicella-zoster virus (VZV) resulted in extensive viral replication, triggering an inflammatory response leading to necrosis within the affected ganglion (7, 8). BP typically presents with unilateral facial weakness, rarely bilateral, and may be accompanied by paresthesia, postauricular pain, hyperacusis, ageusia, or epiphora (9–11). The yearly incidence of BP was estimated to be 24.5 patients per 100,000 population from 2007 to 2022 (12). An article published in 2010 in Italy reported that the cumulative incidence was 53.3/100,000 per year (13). Additionally, researchers have reported an increased incidence during the third and fourth decades of life and inconsistent distribution between males and females (10, 14–17). More recently, a large-scale genetic meta-analysis has found evidence supporting a correlation between BP and a sequence variant, rs9357446-A (18).

One current significant discussion regarding BP is the influence of temperature on its onset. Research findings have indicated a notable link between low temperatures and BP development, suggesting that colder months may correlate with a greater BP incidence (19–22). Conversely, two studies have demonstrated differing results, with BP cases increasing during warmer months, particularly in spring and summer (23, 24). This discrepancy highlights the complexity of seasonal changes in BP incidence. Understanding the seasonal effect on BP occurrence is crucial for guiding preventive strategies and patient education, potentially reducing the overall burden of this unpredictable disease. To date, there are limited comprehensive systematic reviews or meta-analyses that have investigated the effects of seasonal changes on BP frequency. The present study aimed to systematically review and analyze the current literature examining the impact of seasonal variability (winter, spring, summer, and autumn) on the incidence of BP.

## 2 Materials and methods

### 2.1 Protocol and registration

This systematic review and meta-analysis was performed per the Preferred Reporting Items in Systematic Reviews and Meta-analysis (PRISMA) guidelines (25). Furthermore, this study has been registered in the Prospective Register of Systematic Reviews (PROSPERO), with protocol number [CRD42024623519]. Ethical approval was not required because of the study's design.

### 2.2 Search strategy

A comprehensive literature search was conducted to identify studies reporting the distribution of BP across the four seasons. Three electronic databases, PubMed, Google Scholar, and Web of Science, were searched from inception until December 2024. Our search strategy combined broad search keywords and Boolean operators, including the following: (“Bell's palsy” OR “Idiopathic Facial Paralysis” OR “Bell Palsy” OR “Idiopathic Facial Palsy” OR “Idiopathic Facial Nerve Palsy”) AND (season^*^ OR winter OR seasonality OR weather OR “seasonal distribution” OR climate OR summer OR temperature). For Google Scholar, the first seven pages of the results retrieved by this strategy were screened in full. This strategy was employed to ensure that all relevant studies on the seasonal patterns in BP were included, and no filters were applied during the searches.

### 2.3 Eligibility criteria

The inclusion and exclusion criteria were predefined to aid the screening process. The inclusion criteria for this study were as follows: (a) population with established idiopathic BP diagnoses and (b) data on the seasonal distribution of BP cases across the four seasons (winter, spring, summer, and autumn). The exclusion criteria were as follows: (a) studies involving other etiologies of facial paralysis or neurological diseases; (b) seasonal incidence was not reported; (c) different definitions of the months in each season; and (d) inadequate reporting of data. Observational studies were eligible, whereas reviews, clinical trials, and case reports were excluded. Articles published before 2000 were excluded to ensure methodological consistency and relevance to current diagnostic practices and those written in languages other than English were also excluded.

### 2.4 Study selection and quality assessment

The literature obtained from the databases was selected for inclusion in two phases: an initial screening of the title and abstract followed by full-text screening. Two authors (T.A.A. and S.K.A.) conducted the title and abstract screening using Rayyan (26). Subsequently, two authors (B.A.A. and K.A.A.) independently evaluated the full texts of the included articles to determine eligibility according to the predefined criteria. Disagreements were resolved by a third author (B.F.A.). Additionally, a manual search of the reference sections of the included studies was performed. Two authors (B.F.A. and F.S.A.) independently assessed the quality of the included studies via the Newcastle–Ottawa Scale (NOS) (27), with disagreements resolved by (T.A.A.). This scale comprises three subscales: selection, comparability, and outcome. There are 9 entries in the scale, and each entry is assigned 1 point, except for comparability, which is allocated 2 points. The maximum score that can be obtained with the tool is 9. A score of 0–3 is considered poor quality and high risk, whereas a score of 4–6 is considered fair quality and moderate risk. For a study to be considered low risk of bias and high quality, it must have a score of 7 or more.

### 2.5 Data extraction

The articles were divided among six authors (B.F.A., F.S.A., B.A.A., K.A.A., S.K.A., and T.A.A.) for independent data extraction. The extraction sheet comprised the following study characteristics: author, country, publication year, study design, and sample size. Relevant patient data, such as age and sex, were retrieved. Additionally, the frequency of BP cases per season was obtained. Relevant information regarding BP, such as the affected side and HBG, was also obtained. Corresponding authors were contacted regarding missing data, and studies with no response were subsequently excluded.

### 2.6 Statistical analysis

The cohort was described via a narrative synthesis approach. The chi-square homogeneity test was used to identify whether seasonal variation was present among the four groups (winter, spring, summer, and autumn). The meta-analysis was conducted in RStudio Version 1.3.959 via the meta package. A random effects model was applied to compute a summary of effects across studies. Pooled estimates were calculated using raw proportions. Moreover, the inverse variance index was used to assess research heterogeneity (*I*^2^), and *I*^2^ >50% was considered heterogeneous. Additionally, a subgroup forest plot with 95% confidence intervals was used to represent the proportion of BP cases in each study. Funnel plots and Egger's regression test were used to visualize and determine the significance of publication bias using Freeman–Tukey double arcsine transformation. A meta-regression analysis was conducted to examine whether specific covariates influenced the observed effect. Sensitivity analyses were also performed by excluding studies with a high risk of bias and extreme values. A *p-value* < 0.05 was considered statistically significant for all tests.

## 3 Results

### 3.1 Search results

**Figure 1:**
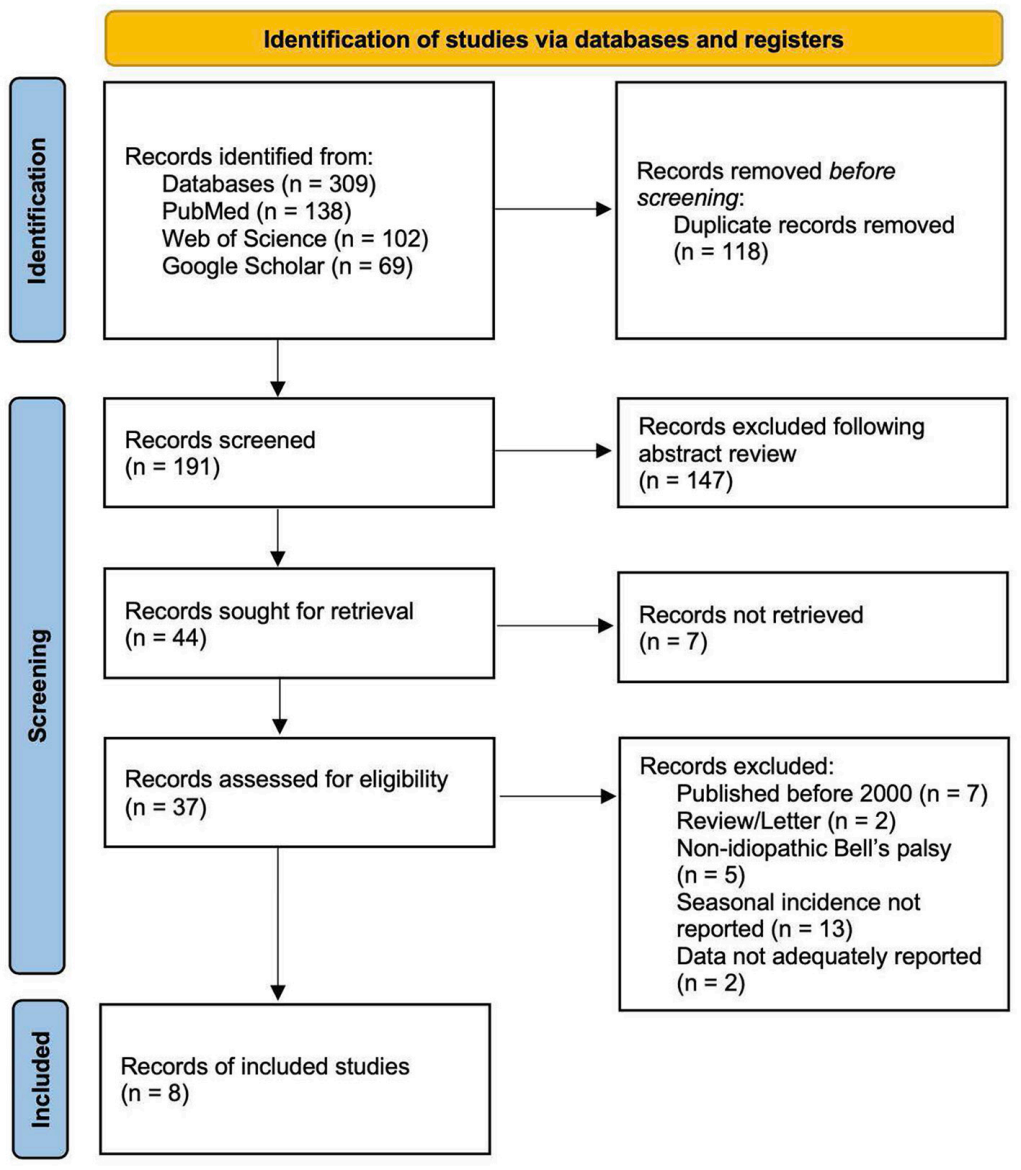
PRISMA 2020 diagram for article screening and selection.

A total of 309 articles were identified from the three databases. The titles and abstracts of 191 articles were screened after 118 duplicates were removed. Following the screening process, 44 articles were retrieved; however, the full texts of seven articles could not be obtained. Thirty-seven articles were assessed for eligibility, 29 of which were excluded per the aforementioned criteria. The reasons for exclusion included that the articles were a review or a letter (*n* = 2), were published before 2000 (*n* = 7), included non-idiopathic BP (*n* = 5), did not report seasonal incidence (*n* = 12), or did not adequately report data (*n* = 2). One study based in Sudan met the inclusion criteria; however, missing data on BP cases in spring were not reported; thus, the study was excluded (24). The detailed PRISMA flow diagram is described in Figure 1.

### 3.2 Patient cohort

Eight cohort studies (19, 21–23, 28–31) were included and evaluated in this systematic review and meta-analysis, encompassing a total of 3,363 patients with BP. Within the population, 1,732 (51.5%) individuals were male, and 1,631 (48.5%) were female. The pooled mean and standard deviation of age among patients with BP were 44.72 ± 19.6 years. Six articles reported the affected side of the paralysis in 1,122 patients, and 50.3% (*n* = 565) had right-sided paralysis (21–23, 28–30). Notably, Danielides et al. (28) reported that females accounted for the greatest percentage of cases of right-side BP (58.7%), whereas 60.7% of the cases of left-side BP were male. A total of 257 patients were evaluated across studies assessing BP severity using the HBG scale. Most cases were graded as HBG III (*n* = 69) or IV (*n* = 70) (21, 29). BP occurrence per season was reported in all studies. Most pooled cases of BP occurred in winter (942 patients; 28%), followed by autumn (863 patients; 25.7%). Furthermore, 842 patients (25%) with BP presented in spring, whereas 716 patients (21.3%) presented in summer. The general characteristics of the included studies are shown in Table 1, while the geographical and climate data are presented in Supplementary Table 1, based on information obtained from the Climate Knowledge Portal (32).

**Table 1:** General characteristics of the included studies.

**Study ID**	**Study design**	**Country**	**Mean Age**	**Sex (M/F)**	**Total**
Yilmaz (2019) (19)	Retrospective	Turkey	42.9	381/435	816
Danielides (2001) (28)	Retrospective	Greece	50.4	62/63	125
Alfaryan (2024) (21)	Retrospective	Saudi Arabia	39.9	79/57	136
Varga (2023) (22)	Retrospective	Hungary	43.02	317/296	613
Kar (2021) (23)	Retrospective	Turkey	42.23	106/93	199
Zohrevandi (2014) (29)	Retrospective	Iran	47.14	64/57	121
Goloom (2021) (30)	Prospective	Iraq	35.6	50/51	101
Spengos (2006) (31)	Retrospective	Greece	47.91	673/579	1,252

(M/F): Male/female.

### 3.3 Test of homogeneity

The chi-square homogeneity test yielded a *p*-*value* < 0.001; thus, a random effects model was used for the meta-analysis to account for study variability (Table 2).

**Table 2:** Chi-square test of homogeneity.

**Study ID**	**Season**				
	**Winter**	**Spring**	**Summer**	**Autumn**	**Total**
Alfaryan (2024) (21)	34	32	27	43	136
Danielides (2001) (28)	29	31	31	34	125
Goloom (2021) (30)	32	27	16	26	101
Kar (2021) (23)	68	41	44	46	199
Spengos (2006) (31)	336	308	268	340	1,252
Varga (2023) (22)	184	177	126	126	613
Yilmaz (2019) (19)	239	210	159	208	816
Zohrevandi (2014) (29)	20	16	45	40	121
	Pearson chi (21) 55.24			*P-value* < 0.001^*^	

^*^P-value significant at < 0.05.

### 3.4 Subgroup analysis

**Figure 2:**
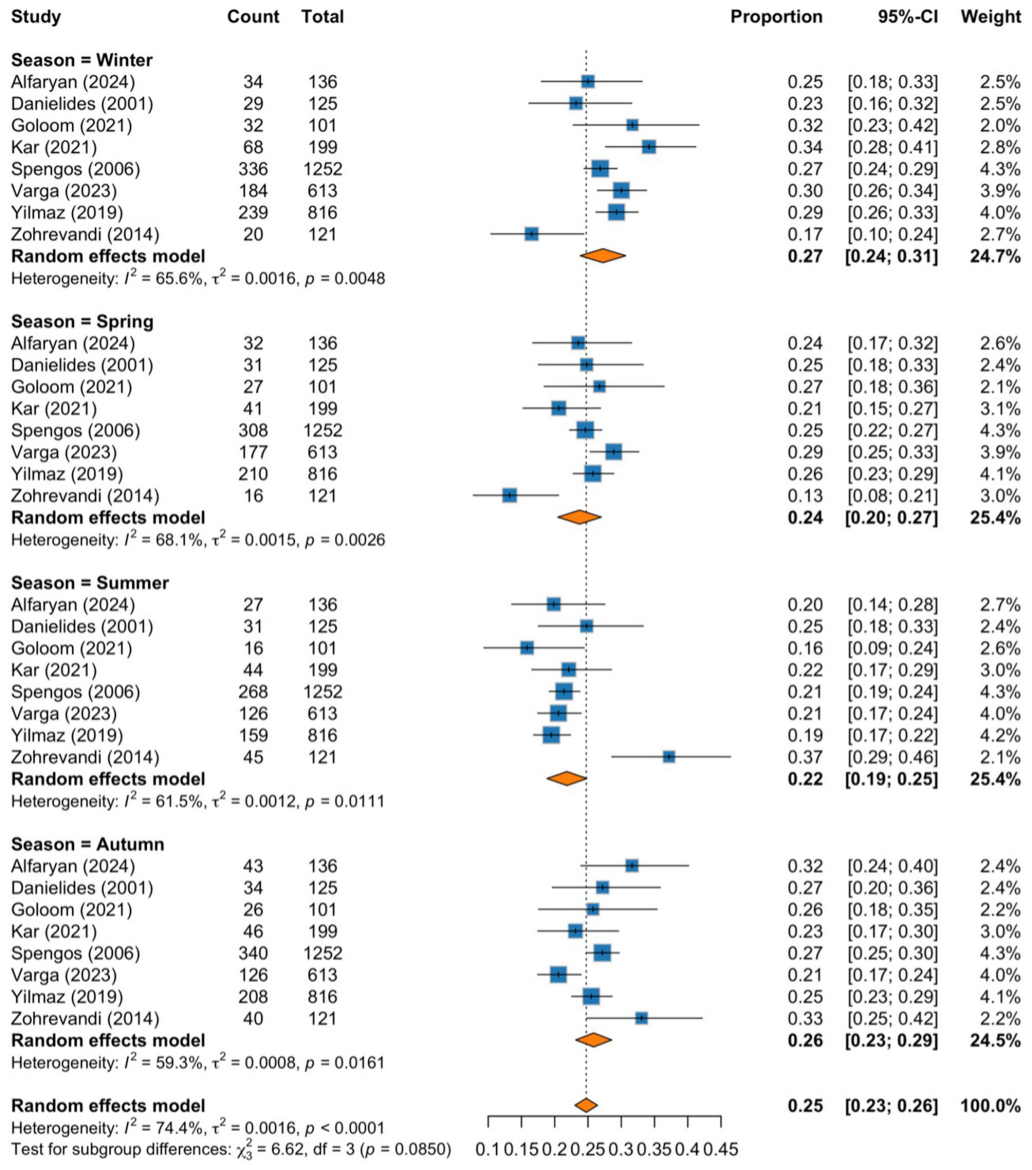
Forest plot of pooled proportions subgrouped by season.

The subgroup analysis shows the pooled proportions of cases for each season, with moderate to substantial heterogeneity observed within each season. The highest pooled proportion of cases was observed during winter, with a proportion of 0.27 (95% CI: 0.24–0.31; *I*^2^ = 65.6%, *p* = 0.0048), followed by autumn at 0.26 (95% CI: 0.23–0.29; *I*^2^ = 59.3%, *p* = 0.0161), spring at 0.24 (95% CI: 0.20–0.27; *I*^2^ = 68.1%, *p* = 0.0026), and the lowest proportion was shown during summer, with a proportion of 0.22 (95% CI: 0.19–0.25; *I*^2^ = 61.5%, *p* = 0.0111). We noticed a seasonal pattern in the occurrence of BP, with colder months showing higher proportions than warmer months. However, the test for subgroup differences revealed a *p-value* of 0.085, indicating a potential trend toward higher incidence in colder seasons (Figure 2).

### 3.5 Publication bias

**Figure 3:**
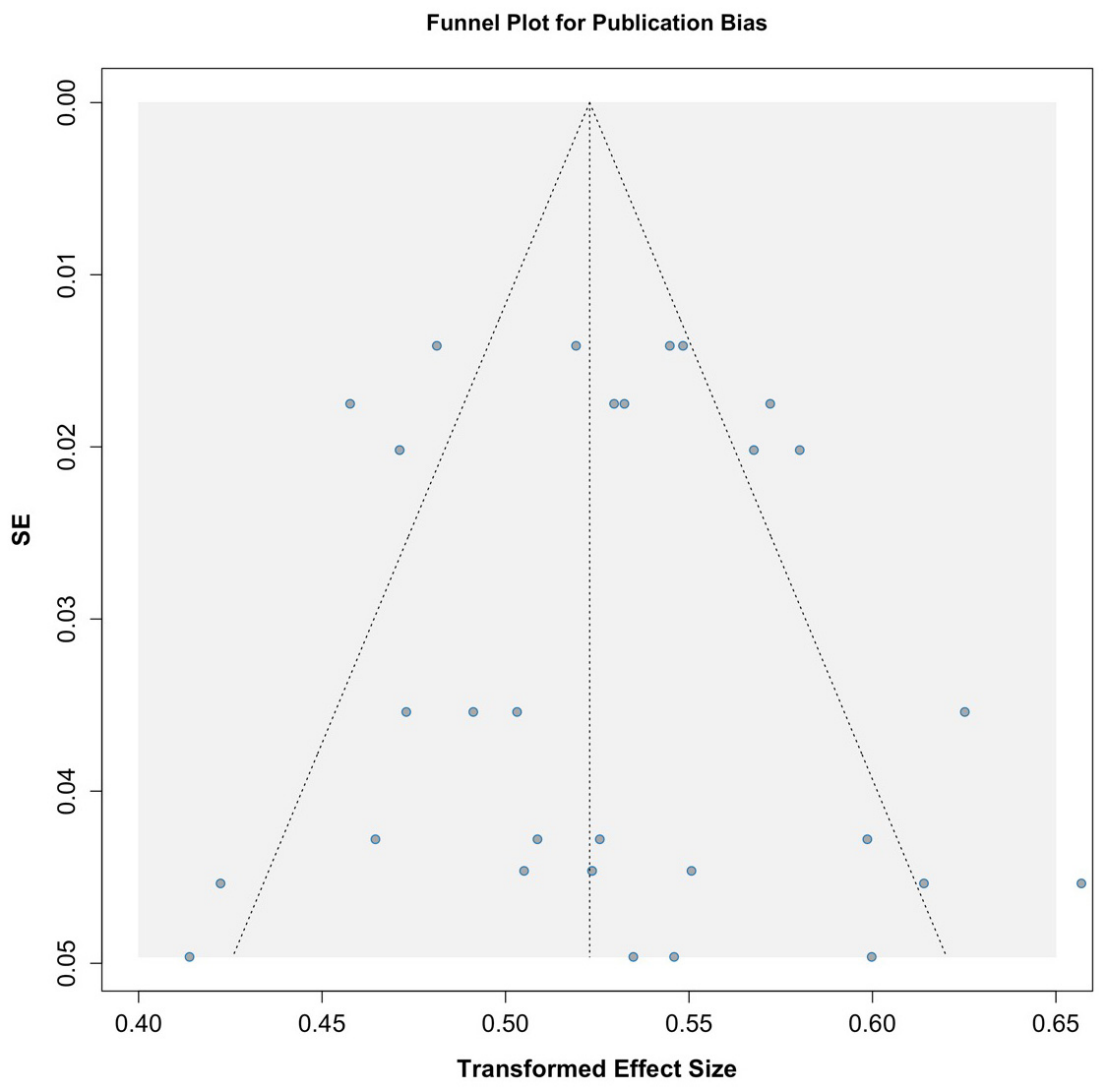
Funnel plot assessing publication bias across included studies.

**Figure 4:**
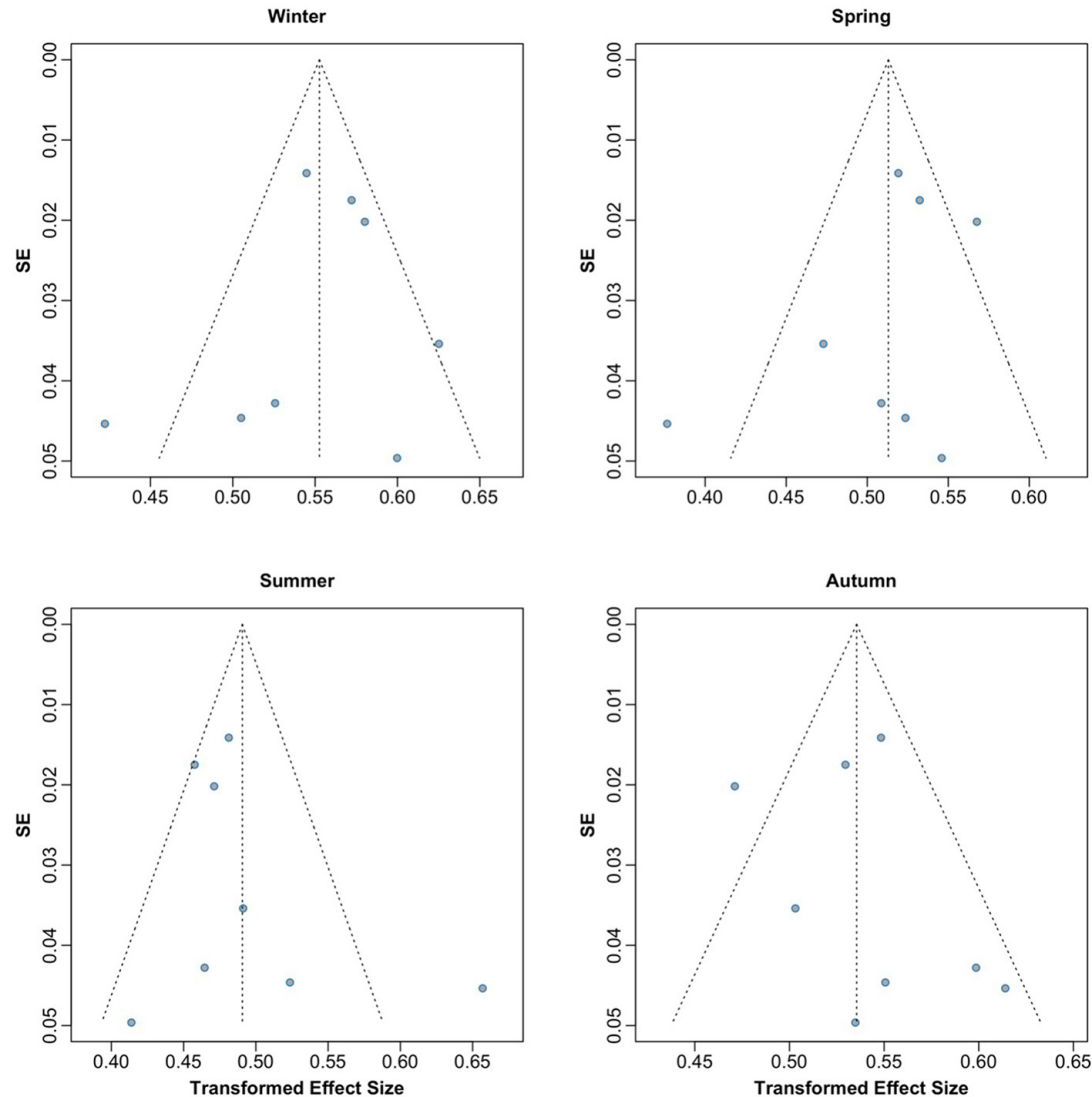
Funnel plots by season assessing potential publication bias across included studies.

Publication bias was assessed using graphical funnel plots with pseudo 95% confidence intervals and Egger's regression test. The funnel plots showed no evidence of publication bias, which was further supported by Egger's regression test (*t* = −0.02, *p* = 0.986; Figure 3). Additionally, analyses stratified by season also revealed no publication bias, with results as follows: winter (*t* = −0.52, *p* = 0.623), spring (*t* = −1.15, *p* = 0.293), summer (*t* = 0.94, *p* = 0.384), and autumn (*t* = 0.62, *p* = 0.557; Figure 4).

### 3.6 Meta-regression analysis

Univariable meta-regression analyses were performed to investigate potential sources of heterogeneity, including region, year of publication, NOS quality score, mean participants age, and sample size. None of the tested covariates were significantly associated with the effect size (*p* > 0.05). These findings indicate that the substantial between-study heterogeneity (*I*^2^ = 74.4%) could not be explained by the available study-level characteristics (Supplementary Table 2).

### 3.7 Quality assessment and risk of bias

Quality assessment via the NOS revealed seven studies (19, 21–23, 28, 29, 31) with a moderate risk of bias (NOS scores of 4–5/9) and one study (30) with a high risk of bias (2/9). Common limitations included a lack of defined exposed groups and inadequate control descriptions. Three studies (22, 28, 29) scored slightly higher (5/9) because of more robust comparability assessments. Notable strengths across studies included representative case selection and standardized outcome assessment methods. The remaining studies (19, 21, 23, 31) scored 4/9, primarily because of single-factor comparability criteria rather than multifactor adjustments (Table 3).

**Table 3:** Literature quality assessment according to Newcastle–Ottawa Scale (NOS).

**Article**	**Selection**			**Comparability**		**Outcome**			**Quality Score**	**ROB^1^**
	**Q1**	**Q2**	**Q3**	**Q4**	**Q5**	**Q6**	**Q7**	**Q8**		
Yilmaz (2019) (19)	^*^	No exposed group	^*^	b) no	^*^	^*^	NA	NA	Fair	Moderate bias
Danielides (2001) (28)	^*^	No exposed group	^*^	b) no	^**^	^*^	NA	NA	Fair	Moderate bias
Alfaryan (2024) (21)	^*^	No exposed group	^*^	b) no	^*^	^*^	NA	NA	Fair	Moderate bias
Varga (2023) (22)	^*^	No exposed group	^*^	b) no	^**^	^*^	NA	NA	Fair	Moderate bias
Kar (2021) (23)	^*^	No exposed group	^*^	b) no	^*^	^*^	NA	NA	Fair	Moderate bias
Zohrevandi (2014) (29)	^*^	No exposed group	^*^	b) no	^**^	^*^	NA	NA	Fair	Moderate bias
Goloom (2021) (30)	^*^	No exposed group	b) no description	b) no	^*^	b) no description	NA	NA	Poor	High bias
Spengos (2006) (31)	^*^	No exposed group	^*^	b) no	^*^	^*^	NA	NA	Fair	Moderate bias

Q1, Representativeness of the Exposed Cohort; Q2, Selection of the Non-Exposed Cohort; Q3, Ascertainment of Exposure; Q4, Demonstration That Outcome of Interest Was Not Present at Start of Study; Q5, Comparability of Cohorts on the Basis of the Design or Analysis; Q6, Assessment of Outcome; Q7, Was Follow-Up Long Enough for Outcomes to Occur; Q8, Adequacy of Follow Up of Cohorts. NA, not applicable. Quality Score: 0–3; poor quality, 4–6; fair quality, 7–9; high quality.

^1^Risk of Bias (0–3; High, 4–6; Moderate, 7–9; Low).

*Denotes to the point for each question as explained in the Methods section - 2.4 Study selection and quality assessment.

### 3.8 Sensitivity analysis

**Figure 5:**
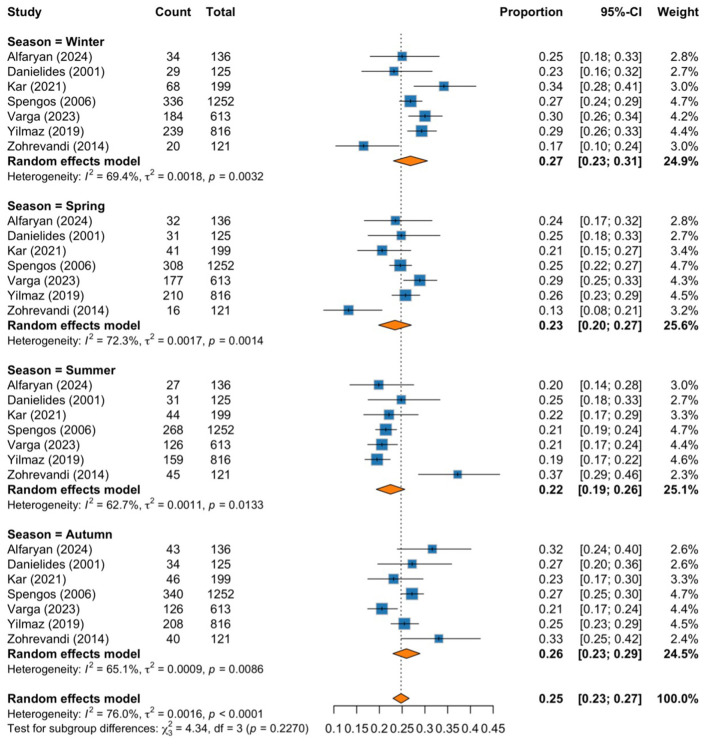
Forest plot subgrouped by season after excluding Goloom et al. (30) (poor-quality study).

Our sensitivity analysis excluded the study by Goloom et al. (30), which received a poor-quality rating on the NOS scale. The exclusion aimed to assess the robustness of the findings and minimize the influence of potential bias from low-quality data. The pooled results remained consistent, suggesting that the overall findings were not meaningfully influenced by this study (Figure 5).

**Figure 6:**
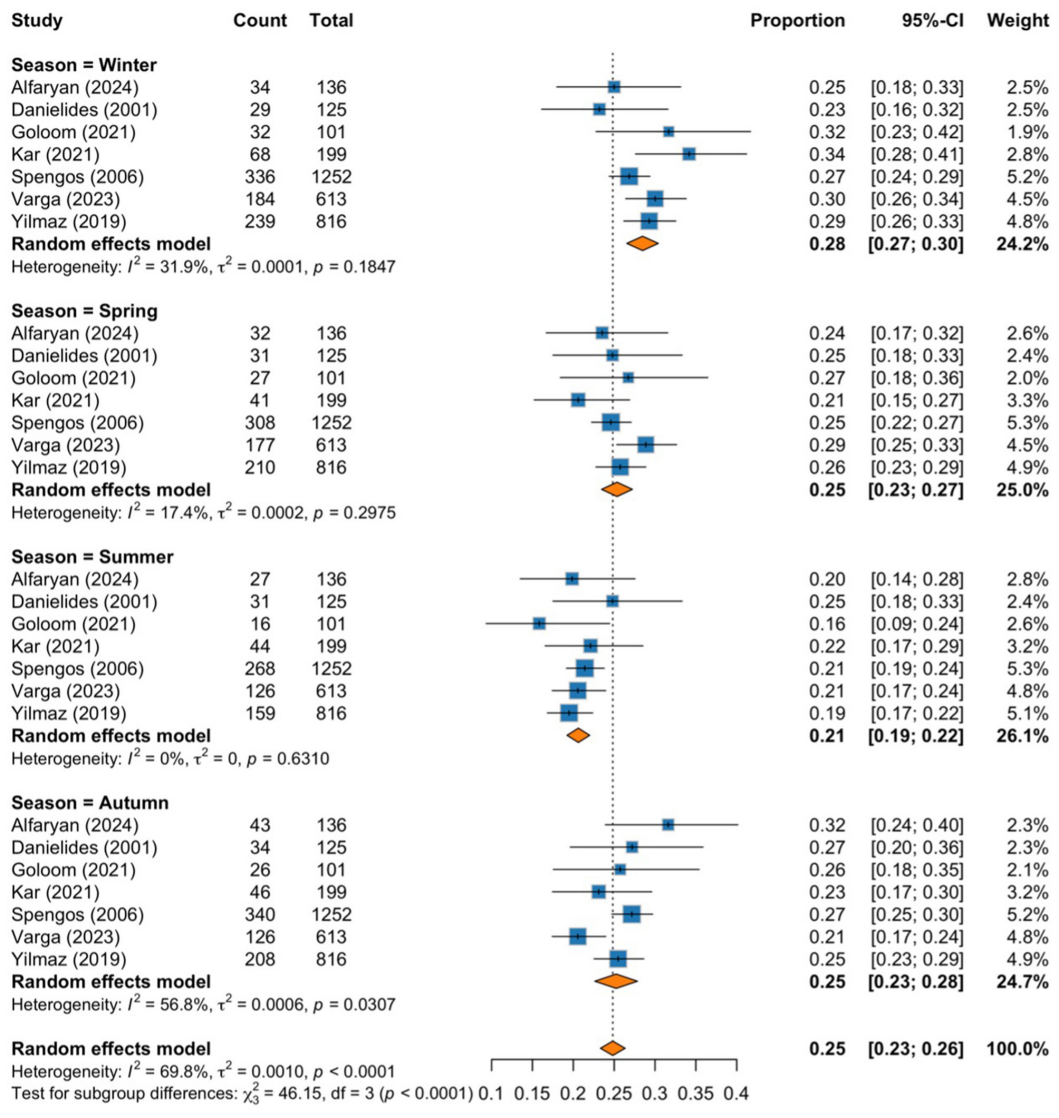
Forest plot subgrouped by season after excluding Zohrevandi et al. (29) (extreme effect sizes).

Also, we excluded the study by Zohrevandi et al. (29), which showed an extreme effect sizes. The exclusion led to a slight reduction in heterogeneity (*I*^2^: from 74.4% to 69.8%), suggesting that this study contributed modestly to between-study variability (Figure 6).

## 4 Discussion

The present study was designed to determine the effect of seasonal variation on the incidence of idiopathic BP across several articles. Researchers in this field have hypothesized that cold weather increases the risk of idiopathic BP development. Thus, multiple studies have been conducted worldwide to test this hypothesis; however, inconsistencies have been reported. Therefore, this systematic review and meta-analysis aimed to consolidate these findings to provide well-supported evidence. This analysis revealed that the highest proportions of BP were encountered during winter (0.27) and the lowest during summer (0.22), reflecting an absolute difference of 5%. The subgroup analysis was found to be statistically insignificant. However, the analysis indicated that there is borderline trend toward higher incidence in cold-weather months.

The pattern observed in colder periods could be explained by vascular and inflammatory factors. Chen and Sun (33) reported that cold exposure increases vasoconstrictor endothelin 1 (ET-1) levels in cardiovascular tissues. Moreover, another study revealed that ET-1 could contribute to BP development by promoting secondary ischemia to the facial nerve (34). Another possible mechanism involves an inflammatory response triggered by upper respiratory tract infections (URTIs), which are commonly encountered during cold months, such as infections with influenza A, respiratory syncytial virus (RSV), or rhinovirus (35). These infections induce the release of inflammatory mediators, which may impair the vascular supply and function of the facial nerve (4).

A similar study employed Google Trends to investigate the seasonal variations of facial paralysis from data across 19 countries, revealing significant peaks in search activity during spring in the northern hemisphere and winter in the southern hemisphere (36). Their different approach offers valuable insight from different perspectives by reflecting public interest in BP. However, this paper is limited in that it solely relies on infodemiological data, unlike our study, which is based on clinical cohort studies of diagnosed BP cases. Our findings contrast with those of previously published studies, which have suggested a significant influence of climate on BP frequency. Zhang et al. (37) published an article in 2024 demonstrating a reduction in daily BP cases of 0.026 (β = −0.026, *p* = 0.003) with each 1 °C increase in temperature in China. Similarly, a study in Greece revealed a strong positive correlation between a composite metric combining temperature and wind speed with BP incidence (Spearman's ρ = 0.406, *p* < 0.001), with cases peaking in the winter months and declining at higher temperatures (38). Additionally, Franzke et al. (39) concluded that the air temperature coupled with the atmospheric pressure are risk factors for BP development. Our findings also align with those of several studies (19, 20, 22–24, 30, 38) showing a peak during winter and others (19–22, 24, 30, 31) demonstrating a decrease in summer. In contrast, three studies (28, 29, 40) demonstrated higher BP cases during the summer and lower proportions during the winter. Five studies (20, 21, 23, 24, 31) demonstrated that, compared with warmer seasons, colder seasons, including autumn and winter, are associated with a greater incidence of BP. Notably, some of the included studies presented inconsistent results compared with other studies conducted in the same region with similar climates according to the Köppen–Geiger climate classification system (41). Moreover, two studies (28, 31) conducted in Greece and two (19, 23) conducted in Turkey revealed inconsistent results despite the almost identical population characteristics and climates. These observations and the pooled effect analysis support our finding of no significant seasonal effect on BP development.

The importance of these findings lies in understanding the patterns of BP. Per our findings, we now know that there is no association between seasonal shifts and the incidence of BP. However, the increased number of cases in winter months may still carry clinical relevance when applied to larger populations, where even small percentage shifts translate into a considerable number of additional cases. This information could be helpful to healthcare providers in alerting at-risk populations and encouraging awareness regarding the disease. On the basis of our results and those of previously published papers, general weather conditions do not affect BP; however, specific climatic parameters, such as atmospheric pressure, wind speed, and acute dramatic temperature changes, might play a role.

This study employed a comprehensive search strategy across multiple databases, adhered to the PRISMA (25) guidelines to ensure high quality and a proven reporting process, and used a unified seasonal definition to minimize categorization bias. In this review (8 studies, *n* = 3,363), we address a notable gap in the literature by systematically evaluating the potential seasonal variation in BP, offering evidence that enhances understanding of its potential epidemiological patterns. Furthermore, the geographic proximity of the included studies strengthens the validity of the findings. Additionally, the funnel plots and Egger's test revealed no publication bias among the included studies, further strengthening our findings' validity. Despite these strengths, this study has several limitations. First, pooled estimates were calculated using raw proportions without variance-stabilizing transformation, which may introduce minor distributional bias. Second, significant heterogeneity was observed across studies (*I*^2^ = 74.4%). This heterogeneity could negatively impact the results if it is due to variability in population characteristics or geographical location, potentially affecting the accuracy and generalizability of our findings. Conversely, the heterogeneity could have arisen from variability in the reported proportions, which would be expected if the seasonal changes had no influence. Nonetheless, heterogeneity was accounted for using a random effects model and subgroup analysis, as well as testing for publication bias. Third, the limited number of included studies (*n* = 8) may have reduced the statistical power to detect true seasonal variations. Moreover, the results of Egger's regression test should be interpreted with caution, as the test has limited statistical power in meta-analyses that include fewer studies. With only eight studies in this analysis, the ability to detect small-study effects or publication bias may be reduced. Fourth, most of the included studies were conducted in regions that are part of or close to the Middle East, the Mediterranean Sea, and with limited representation from European regions. This restricted geographical coverage limits the generalizability of our results to global seasonal patterns and biases them toward no global seasonality. Fifth, meta-regression analyses did not identify any study-level characteristics (such as region, publication year, NOS quality rating, mean participants' age, or sample size) that significantly contributed to the observed heterogeneity. This suggests that unmeasured factors, such as methodological differences, population characteristics, or contextual factors (e.g., healthcare systems, climate, or diagnostic criteria), may account for the variability between studies. Sixth, the NOS evaluation revealed that the included studies had a high-to-moderate risk of bias and low-to-fair quality but to a degree that was insufficient to warrant their exclusion, and no inter-rater agreement metrics were assessed, which may have introduced subjectivity in the quality assessment. Another limitation is that the exclusion of studies published before 2000 and non-English publications may have introduced selection bias by omitting otherwise eligible studies. Additionally, none of the studies provided a detailed breakdown of HBG severity scores for each season, limiting our ability to assess potential seasonal influences on BP severity. Finally, risk factors and clinical status, such as the affected side, previous infections, and comorbidities, were not considered when the seasonal impact on BP was analyzed, as these factors were also not provided by the included studies for each season.

Future studies, preferably longitudinal studies, should be undertaken to confirm whether there is a link between specific meteorological parameters and BP and to clarify whether seasonal patterns differ by age, sex, or comorbidities, such as hypertension, diabetes mellitus, and immunity status, which could reveal additional information regarding the underlying etiology of this disease. Furthermore, future meta-analyses including a larger number of studies are warranted to confirm these findings.

## 5 Conclusion

This systematic review and meta-analysis investigated the association between the four seasons and BP occurrence. No statistically significant difference across seasons on the risk of developing BP has been found. However, the highest proportion of BP cases occurred during winter, followed by autumn, spring, and summer. This observation promotes targeted patient education during colder months, emphasizing recognition of BP symptoms to encourage earlier presentation and timely management. Because the studies included in this review were conducted in limited number of geographical locations, further epidemiological research is needed across diverse climatic regions using specific climatic parameters.

## Data Availability

The original contributions presented in the study are included in the article/Supplementary material, further inquiries can be directed to the corresponding author.
