# Supplementary material for: Seasonal patterns in Bell's palsy: a systematic review and meta-analysis
Source: Front Neurol. 2025 Nov 11;16:1626018. doi: 10.3389/fneur.2025.1626018 (PMC12643973; doi:10.3389/fneur.2025.1626018)
Supplement: Supplementary file 1 [file Table_1.docx]

Supplementary Table 1: Average seasonal temperatures based on the geographical locations of the included studies

| **Study** | **Country** | **Winter** | **Spring** | **Summer** | **Autumn** | **Avg. Winter Temp (°C)** | **Avg. Spring Temp (°C)** | **Avg. Summer Temp (°C**) | **Avg. Autumn Temp (°C)** |
| --- | --- | --- | --- | --- | --- | --- | --- | --- | --- |
| Yilmaz (2019) [19] | Turkey | 239 | 210 | 159 | 208 | 4.72 | 11.91 | 22.89 | 15.32 |
| Danielides (2001) [28] | Greece | 29 | 31 | 31 | 34 | 9.21 | 14.43 | 24.52 | 17.72 |
| Alfaryan (2024) [21] | Saudi Arabia | 34 | 32 | 27 | 43 | 16.59 | 27.21 | 35.24 | 26.9 |
| Varga (2023) [22] | Hungary | 184 | 177 | 126 | 126 | 0.29 | 11.6 | 21.52 | 11.59 |
| Kar (2021) [23] | Turkey | 68 | 41 | 44 | 46 | 4.72 | 11.91 | 22.89 | 15.32 |
| Zohrevandi (2014) [29] | Iran | 20 | 16 | 45 | 40 | 4.13 | 12.16 | 23.8 | 15.32 |
| Goloom (2021) [30] | Iraq | 32 | 27 | 16 | 26 | 12.14 | 24.3 | 35.45 | 25.44 |
| Spengos (2006) [31] | Greece | 336 | 308 | 268 | 340 | 9.01 | 14.81 | 25.66 | 18.09 |

Avg.: Average; Temp: Temperature.
