# Supplementary material for: Seasonal patterns in Bell's palsy: a systematic review and meta-analysis
Source: Front Neurol. 2025 Nov 11;16:1626018. doi: 10.3389/fneur.2025.1626018 (PMC12643973; doi:10.3389/fneur.2025.1626018)
Supplement: Supplementary file 2 [file Table_2.docx]

Supplementary Table 2: Univariable meta-regression analyses exploring sources of heterogeneity

| **Moderator Variable** | **β** | **SE** | **95% CI**  **(Lower, Upper)** | **p-value** |
| --- | --- | --- | --- | --- |
| Region  *Hungary vs. Greece*  *Iran vs. Greece*  *Iraq vs. Greece*  *Saudi Arabia vs. Greece*  *Turkey vs. Greece* | -0.0017  -0.0068  -0.0006  0.0002  -0.0012 | 0.0306  0.0367  0.0381  0.0359  0.0260 | (-0.0616, 0.0582)  (-0.0787, 0.0652)  (-0.0752, 0.0740)  (-0.0703, 0.0706)  (-0.0522, 0.0498) | 0.9558  0.8539  0.9871  0.9964  0.9629 |
| NOS quality score  *Poor vs. Fair* | 0.0007 | 0.0347 | (-0.0673, 0.0688) | 0.9829 |
| Year of publication | -0.0000 | 0.0013 | (-0.0025, 0.0024) | 0.9714 |
| Mean participants age | -0.0000 | 0.0024 | (-0.0048, 0.0047) | 0.9869 |
| Sample size | 0.0000 | 0.0000 | (-0.0000, 0.0000) | 0.9868 |

*Note: β =Coefficient (Estimate); SE= Standerd Error; CI= Confidence Interval; NOS= Newcastle-Ottawa Scale.*
